# Multiparametric CEST and Z-spectrum analysis proton (ZAP) as biomarkers of human brain aging

**DOI:** 10.1038/s41598-026-46623-6

**Published:** 2026-04-04

**Authors:** Hye Na Jung, Vadim Malis, Mitsue Miyazaki

**Affiliations:** 1 Department of Radiology, Korea University Guro Hospital, Korea University College of Medicine, Seoul, Republic of Korea; 2 Department of Radiology, University of California San Diego, San Diego, CA USA

**Keywords:** Magnetization transfer MRI, Chemical exchange saturation transfer (CEST), Z-spectrum analysis protons (ZAP), Aging, Neurodegeneration, Biomarkers, Neurology, Neuroscience

## Abstract

Chemical exchange saturation transfer (CEST) has been investigated to capture specific hydroxyl, amine, and amide protons. Z-spectrum analysis protons (ZAP) technique quantifies relaxation times of relatively free- ($\:{T}_{\:\:2,f}^{ex}$) and restricted exchange proton ($\:{T}_{\:\:2,r}^{ex}$ ) pools, and their fractions (F_f_ and F_r_) from a wider frequency range. Aging is related to brain neurodegeneration. However, the relationship between brain aging and macromolecular exchange protons remains unclear. Here we investigated brain aging by combining CEST and ZAP for comprehensive assessment of the macromolecules as potential biomarkers. Forty-three healthy adults (65.6 ± 16.0 years; 24 females) underwent 3-T experiments. Gray matter (GM), white matter (WM), and cerebrospinal fluid (CSF) were automatically segmented, and regions of interest were drawn in hippocampus, substantia nigra, globus pallidus, putamen, caudate, and thalamus. Spearman’s rank correlation coefficient analysis revealed significant age-related increases in F_f_ across WM, CSF, hippocampus, substantia nigra, globus pallidus, and putamen (all *P* < 0.05), along with elevated $\:{T}_{\:\:2,f}^{ex}$ in the cortical GM, WM, and thalamus (all *P* < 0.05). Amine signal in CSF decreased with age (*P* = 0.049). These results suggest age-related macromolecular alterations, highlighting CEST-ZAP metrics as promising biomarkers for brain aging and neurodegeneration.

**Supplementary Information:**

The online version contains supplementary material available at 10.1038/s41598-026-46623-6.

## Introduction

Magnetization transfer (MT) contrast provides indirect information on macromolecular content by exploiting magnetization exchange between immobile semi-solid macromolecular protons and free water protons^1^. This approach enhances tissue contrast and has also been useful in assessing structural alterations related to normal aging or pathologic tissues^2–8^. Chemical exchange saturation transfer (CEST) magnetic resonance imaging (MRI) is an emerging molecular imaging tool to differentiate between healthy and pathological condition based on the presence of CEST exchange protons and their amounts. The CEST technique detects hydroxyl (—OH, ∼1.0 ppm), amine (—NH_2_, ∼2 ppm), and amide (—NH, ∼3.5 ppm). In particular, amide proton transfer (APT) weighted-signal intensity is elevated in malignant brain tissues^9^, and APT-CEST has been proposed as a predictive tool for prognosis of glioma^10^ as well as useful technique for detecting and grading brain tumors^11^. In addition to these oncologic applications, CEST MRI allows noninvasive, quantitative assessment of endogenous and exogenous metabolites and subtle tissue microenvironmental alterations^12–14^, thereby providing advanced metabolic and microstructural characterization that complements and extends conventional MRI.

**Fig. 1:**
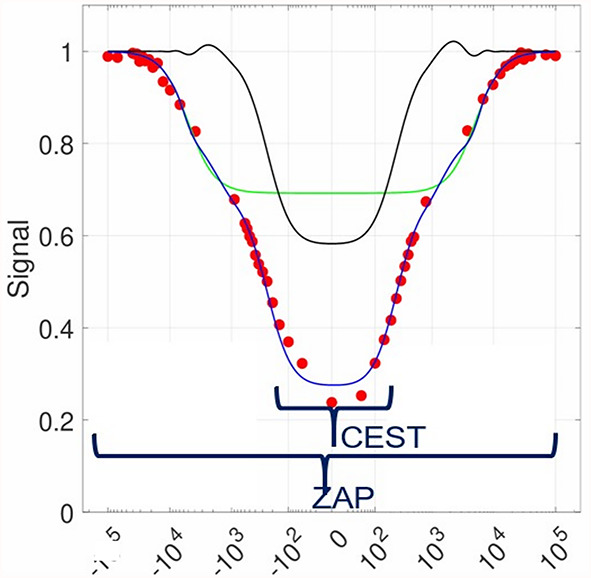
An example of Z-spectrum in logarithm. Chemical exchange saturation transfer (CEST) range and entire Z-spectrum analysis protons (ZAP) range. Red dots indicate actual measured signal intensities. Black fitting shows apparent relaxation time of relatively free water proton ($\:{T}_{\:\:2,f}^{ex}$) and green indicates apparent relaxation time of relatively restricted water proton ($\:{T}_{\:\:2,r}^{ex}$). Blue fitting is combined black and green fits.

CEST MRI provides sensitivity to low-concentration endogenous metabolites and mobile proteins through selective saturation of bound protons that exchange with bulk water protons^1^. The resulting decrease in water signal is plotted against frequency offset, yielding a Z-spectrum, allowing for frequency-specific analysis of exchange processes (− 1 to 1 kHz or ± 8 ppm) using the MT ratio asymmetry (MTR_Asym_) approach^1,15^. However, because the CEST signal in MTR_Asym_ is typically only 2–4%, the resulting low sensitivity makes it challenging to differentiate normal from diseased tissue. In contrast, Z-spectrum analysis protons (ZAP) utilizes entire spectrum of frequencies (− 100 to 100 kHz or ± 800 ppm) and provides metrics that quantify entire spectrum of exchange protons^16^. ZAP employes a two-compartment Lorentzian model to estimate the apparent relaxation times of relatively free- and restricted (bound) exchangeable water protons ($\:{T}_{\:\:2,f}^{ex}$ and $T_{2, r}^{ex}$ , respectively), and their fractions (F_f_ and F_r,_ respectively)^15,16^. The presence of ultra-short T2 exchange protons, as $T_{2, r}^{ex}$ in ZAP, is first described by the super-Lorentzian lineshape using a two-pool model^17^. Our $T_{2, r}^{ex}$ values are in the order of a couple of ten microseconds, which are similar to their restricted proton values^17^. To precise measurement of $\:{T}_{\:\:2,f}^{ex}$, $T_{2, r}^{ex}$ , and their fractions, we acquired entire wide range of frequency and analyzed using the two-Lorentzian Z-spectrum fitting with only three independent variables^15,16^. Figure 1 shows an example of Z-spectrum in log-modulus scale containing CEST and ZAP frequency ranges with actual measured signal intensities denoted with red markers.

The ZAP frequency range contains the CEST frequency range. Combing both CEST and ZAP can reveal regional presence of specific protons such as hydroxyl (—OH), amine (—NH_2_), and amide (—NH) proton which are hidden under the Z-spectrum as well as entire exchange protons. These combined CEST and ZAP approach provides comprehensive assessment of the macromolecular exchanges in specific CEST protons and quantification of relatively free- and restricted exchange proton pools.

Age-related changes in brain tissue may alter macromolecular structure, concentration, and proton exchange dynamics. The aim of this study is to apply the previously proposed and validated combined ZAP and CEST acquisition and analysis framework^15^ to evaluate its sensitivity to age-related changes and the potential of this multiparametric approach to establish biomarkers of brain aging.

## Results

**Fig. 2:**
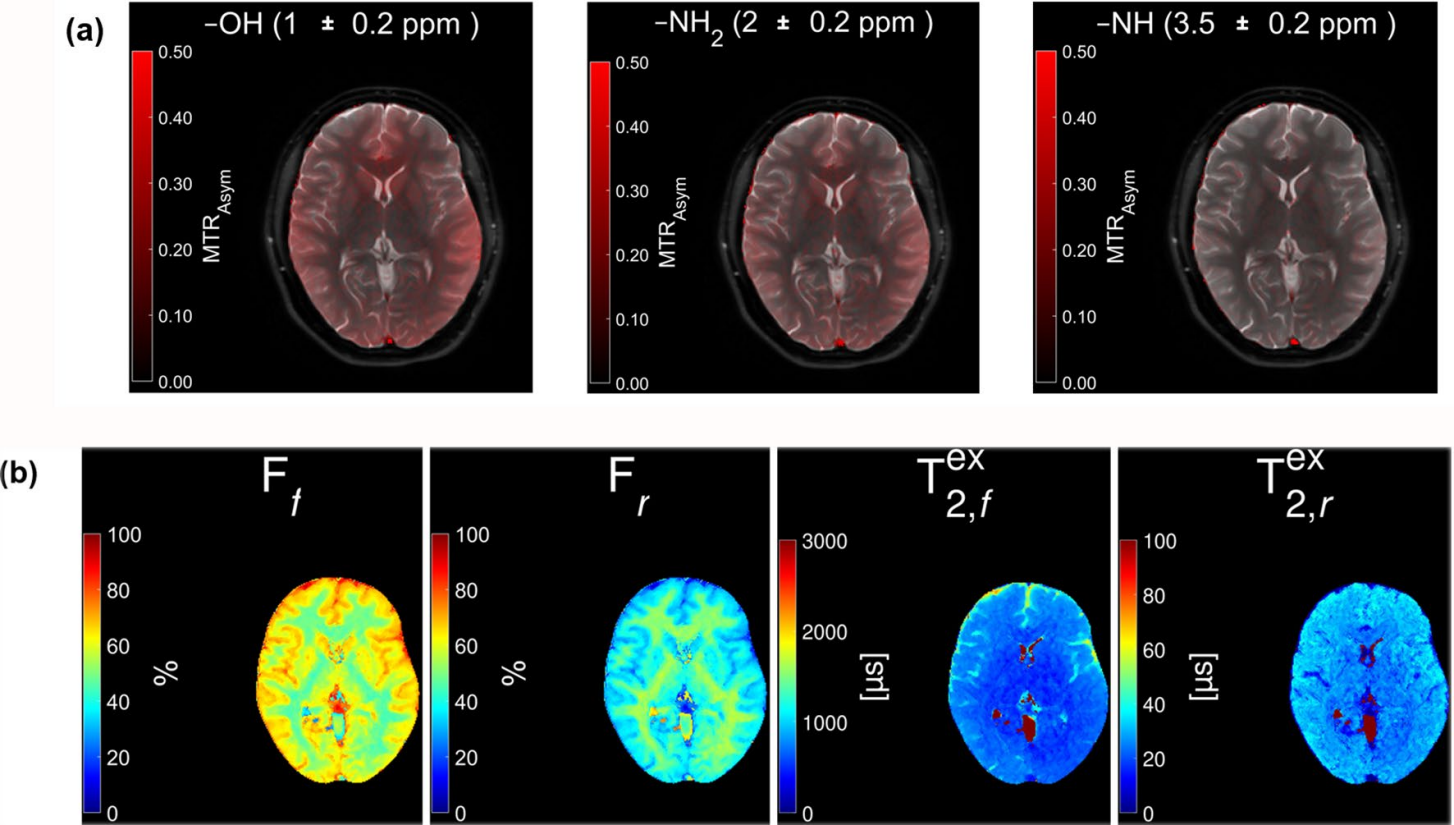
Colormaps of the metrics extracted from the chemical exchange saturation transfer (CEST) / Z-spectrum analysis protons (ZAP) analysis for a participant. CEST (—OH, —NH2, —NH) images (a) & ZAP (F_f_, F_r_, $\:{T}_{\:\:2,f}^{ex}$ and $\:{T}_{\:\:2,r}^{ex}$) fusion images (b). F_f_ fraction of relatively free water proton, F_r_ fraction of relatively restricted water proton, $\:{T}_{\:\:2,f}^{ex}$ apparent relaxation time of relatively free water proton, $\:{T}_{\:\:2,r}^{ex}$ apparent relaxation time of relatively restricted water proton.

The white matter (WM), gray matter (GM), and cerebrospinal fluid (CSF) were successfully identified and segmented for all participants. Figure 2 presents color maps of CEST protons (2a), and ZAP metrics in $\:{T}_{\:\:2,f}^{ex}$, $T_{2, r}^{ex}$, and their fractions, F_f_ and F_r_, respectively (2b). Note that F_f_ and F_r_ =1[16].

### ZAP metrics

**Fig. 3:**
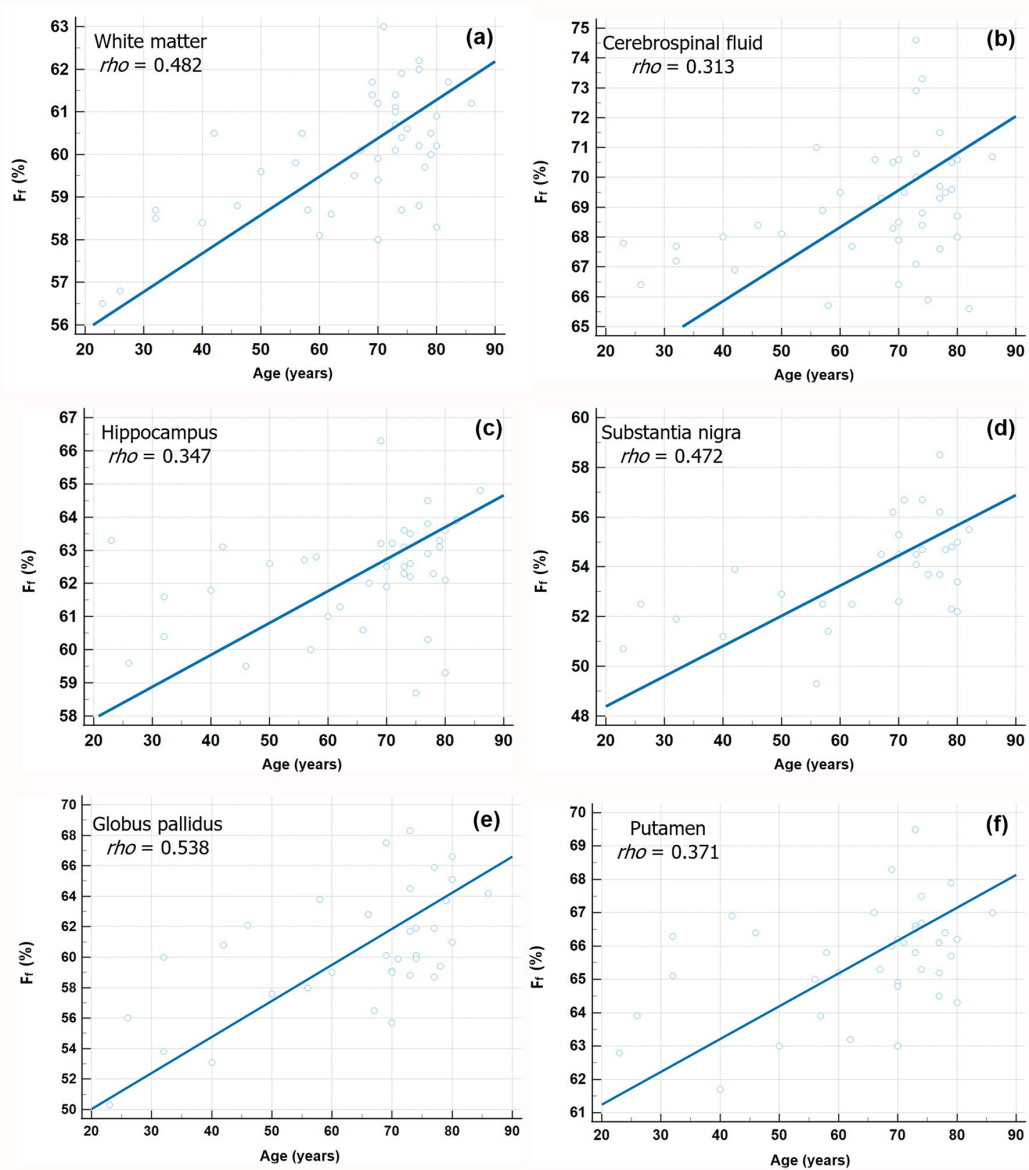
Correlation of F_f_ with age across brain subregions. Each blue circle represents the age of each participant (x-axis) and the mean F_f_ (y-axis) within a brain subregion. The blue linear line shows the relation between the corresponding F_f_ values and ages (a-f). Note: only regions showing statistically significant correlations (*P* values < 0.05) are displayed. F_f_ fraction of relatively free water proton, *rho* Spearman’s rank correlation coefficient.

**Fig. 4:**
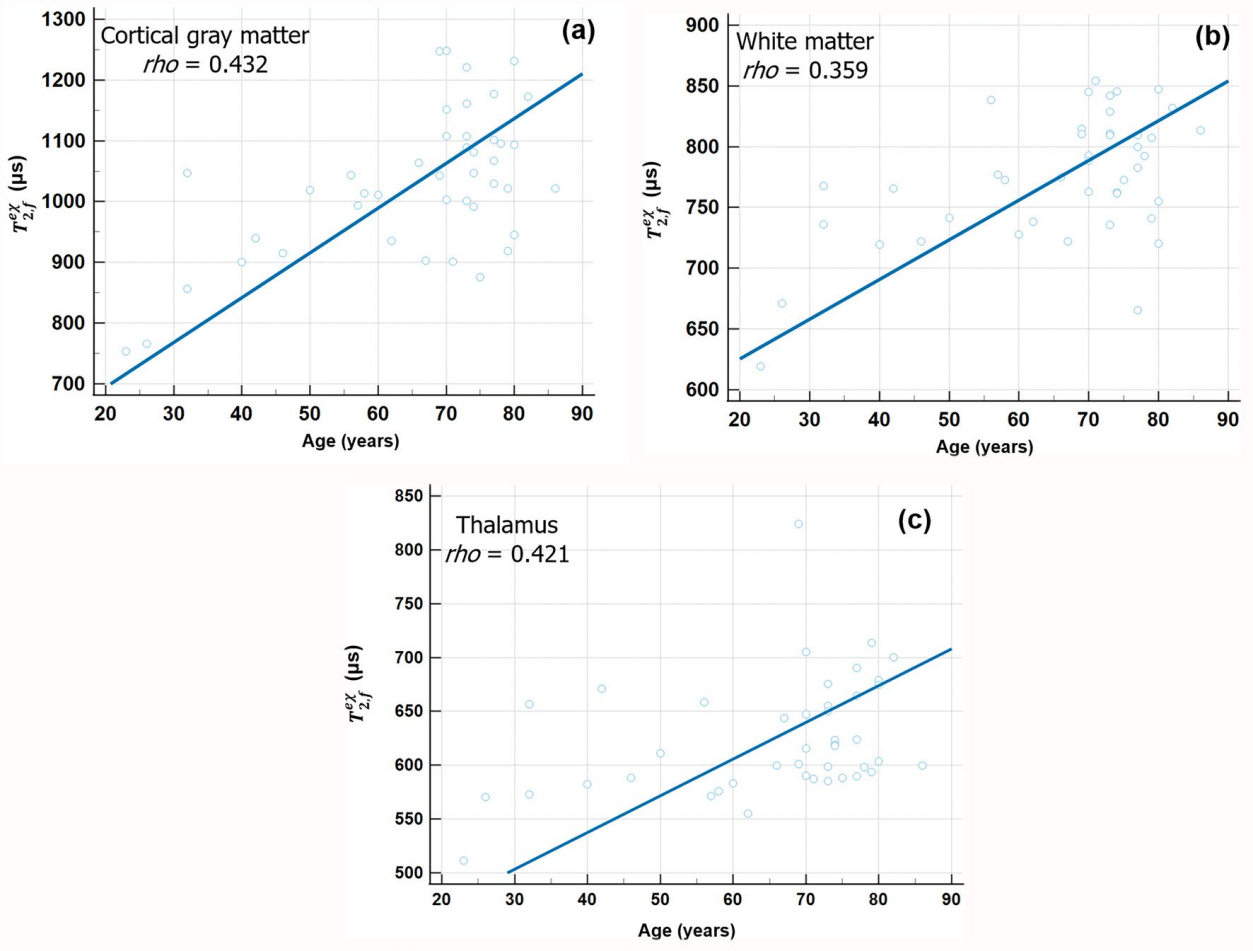
Correlation of $\:{T}_{\:\:2,f}^{ex}$ with age across brain subregions. Each blue circle represents the age of each participant (x-axis) and the mean $\:{T}_{\:\:2,f}^{ex}$ (y-axis) within a brain subregion. The blue linear line shows the relation between the corresponding $\:{T}_{\:\:2,f}^{ex}$ values and ages (a-c). Note: only regions showing statistically significant correlations (*P* values < 0.05) are displayed. $\:{T}_{\:\:2,f}^{ex}$ apparent relaxation time of relatively free water proton, *rho* Spearman’s rank correlation coefficient.

**Fig. 5:**
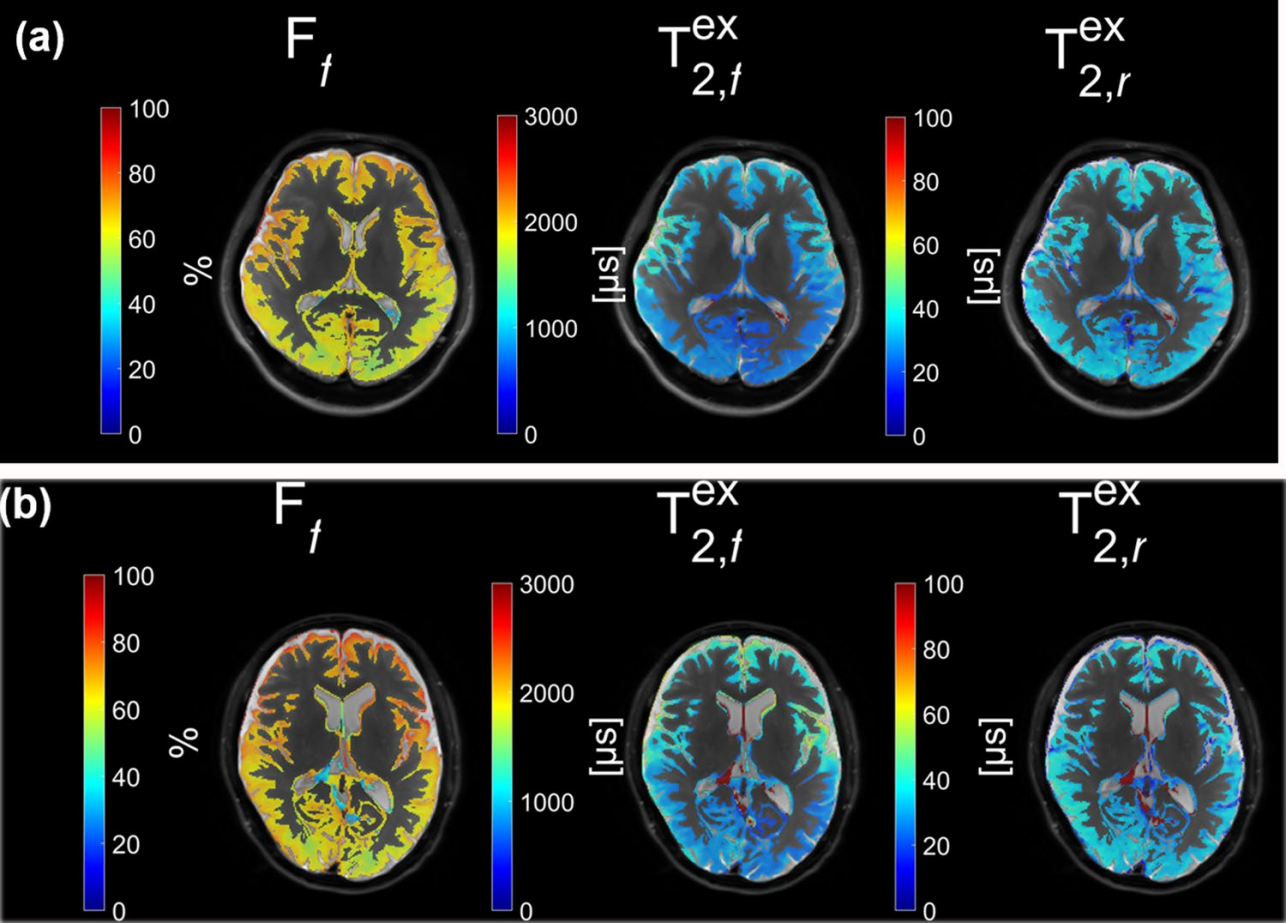
Representative cases revealing correlation of ZAP metrics with age in cortical gray matter (GM). Fraction of relatively free water proton (F_f_), apparent relaxation time of relatively free- ($\:{T}_{\:\:2,f}^{ex}$) and restricted ($\:{T}_{\:\:2,r}^{ex}$) water proton was 63.8%, 900.6 μs and 36.4 μs in a 40-year-old male (a), and 66.4%, 1081.6 μs and 40.5 μs in a 74-year-old male (b), respectively. Representative colormap images of the cortical GM illustrate a relatively thinner cortex in the older participant (b) compared with the younger participant (a).

Based on ZAP metrics, aging was associated with an increased F_f_ (%) in the WM {*rho =* 0.482; 95% confidence interval (CI), 0.213–0.684; *P* = 0.001}, CSF (*rho =* 0.313; 95% CI, 0.0142–0.561; *P* = 0.041), hippocampus (*rho =* 0.347; 95% CI, 0.053–0.587; *P* = 0.023), substantial nigra (SN) (*rho =* 0.472; 95% CI, 0.153–0.701; *P* = 0.006), globus pallidus (GP) (*rho =* 0.538; 95% CI, 0.250–0.739; *P* < 0.001), and putamen (*rho =* 0.371; 95% CI, 0.058–0.617; *P* = 0.022) statistically (Fig. 3). Spearman’s rank correlation coefficient also revealed positive correlation between age and $\:{T}_{\:\:2,f}^{ex}$ (µs) in the cortical GM (*rho =* 0.432; 95% CI, 0.152–0.648; *P* = 0.004), WM (*rho =* 0.359; 95% CI, 0.066–0.595; *P* = 0.018), and thalamus (*rho =* 0.421; 95% CI, 0.138–0.640; *P* = 0.005) (Fig. 4). In terms of $T_{2, r}^{ex}$ (µs), there is no significant correlation between age and any subregion of the brain. Table 1 summarizes all Spearman’s rank correlation coefficient (*rho*) between participants’ age and ZAP metrics in multiple anatomic structures. Significant differences in F_f_ value*s* were observed across all brain subregions between younger and older adults groups (all *P* ≤ 0.025) (Table 2). For $\:{T}_{\:\:2,f}^{ex}$, cortical GM, WM, CSF, and thalamus exhibited significant differences between the two groups (all *P* ≤ 0.008). In contrast, no statistically significant differences were detected between the groups in the $T_{2, r}^{ex}$ (all *P* > 0.05). These findings are summarized in Table 3. Figure 5 shows representative cases.

**Table 1:** Spearman’s rank correlation coefficient (*rho*) between participants’ age and Z-spectrum analysis protons metrics in multiple anatomic structures.

Brain structure	F_f_ (%)	$\:{T}_{\:\:2,f}^{ex}$ (μs)	$\:{T}_{\:\:2,r}^{ex}$ (µs)
Cortical GM (*n* = 43)	0.286 (0.063)	**0.432 (0.004)**	0.200 (0.198)
WM (*n* = 43)	**0.482 (0.001)**	**0.359 (0.018)**	0.112 (0.476)
CSF (*n* = 43)	**0.313 (0.041)**	0.238 (0.125)	0.151 (0.335)
Hippocampus (*n* = 43)	**0.347 (0.023)**	0.044 (0.778)	−0.023 (0.883)
Substantia nigra (*n* = 33)	**0.472 (0.006)**	0.075 (0.677)	−0.038 (0.834)
Globus pallidus (*n* = 35)	**0.538 (< 0.001)**	0.106 (0.544)	0.079 (0.652)
Putamen (*n* = 38)	**0.371 (0.022)**	0.266 (0.107)	0.118 (0.480)
Caudate (*n* = 43)	0.297 (0.053)	0.129 (0.409)	0.052 (0.743)
Thalamus (*n* = 43)	0.276 (0.073)	**0.421 (0.005)**	0.183 (0.241)

Note: Numbers represent Spearman’s rank correlation coefficient. Numbers in parentheses denote the *P*-values; *P* < 0.05 are displayed in **bold**. F_f_ fraction of relatively free water proton, $\:{T}_{\:\:2,f}^{ex}$ apparent relaxation time of relatively free water proton; $\:{T}_{\:\:2,r}^{ex}$ apparent relaxation time of relatively restricted water proton.

**Table 2:** Comparison of mean values (standard deviation) of F_f_ (%) between younger (< 65 years, *n* = 13) and older (≥ 65 years, *n* = 30) adult groups in multiple regions of interest.

Brain structure	Younger	Older	*P* value
Cortical GM	64.31 (0.85)	65.26 (1.20)	**0.013**
WM	58.73 (1.21)	60.53 (1.19)	**< 0.001**
CSF	67.95 (1.36)	69.49 (2.05)	**0.017**
Hippocampus	61.52 (1.34)	62.65 (1.52)	**0.025**
Substantia nigra^*^	51.88 (1.29)	54.80 (1.52)	**< 0.001**
Globus pallidus^†^	57.68 (4.09)	61.76 (3.37)	**0.004**
Putamen^§^	64.55 (1.60)	66.11 (1.37)	**0.003**
Caudate	65.88 (1.26)	67.76 (2.19)	**0.001**
Thalamus	54.65 (1.37)	56.41 (2.70)	**0.007**

Note: ^*^Younger (*n* = 10), older (*n* = 23); ^†^Younger (*n* = 11), older (*n* = 24); ^§^ Younger (*n* = 13), older (*n* = 25); *P* < 0.05 are displayed in **bold.** F_f_ fraction of relatively free water proton.

**Table 3:** Comparison of mean values (standard deviation) of $\:{T}_{\:\:2,f}^{ex}$ and $\:{T}_{\:\:2,r}^{ex}$ between younger (< 65 years, *n* = 13) and older (≥ 65 years, *n* = 30) adult groups in multiple regions of interest.

Brain structure	Younger	Older	*P* value
$$\:{T}_{\:\:2,f}^{ex}$$ (μs)			
Cortical GM	943.46 (100.13)	1070.66 (103.47)	**< 0.001**
WM	738.23 (53.07)	790.04 (44.36)	**0.002**
CSF	1944.42 (450.21)	2292.13 (300.55)	**0.005**
Hippocampus^*^	648.34 (616.26–662.16)	657.53 (619.14–708.27)	0.278
Substantia nigra^†^	570.30 (49.39)	563.41 (43.72)	0.692
Globus pallidus^*‡^	574.65 (523.57–610.29)	591.61 (568.61–613.35)	0.334
Putamen^§^	659.37 (48.57)	674.52 (42.56)	0.328
Caudate	726.23 (83.82)	780.22 (91.55)	0.076
Thalamus^*^	583.38 (572.32–640.21)	621.18 (598.56–674.50)	**0.008**
$$\:{T}_{\:\:2,r}^{ex}$$(µs)			
Cortical GM	37.60 (4.00)	39.95 (4.02)	0.084
WM	30.19 (2.15)	31.32 (2.59)	0.176
CSF	87.18 (29.56)	101.94 (28.28)	0.129
Hippocampus	28.87 (1.36)	29.00 (2.49)	0.827
Substantia nigra^†^	25.75 (2.08)	25.21 (1.86)	0.466
Globus pallidus^*‡^	27.20 (25.56–27.72)	27.59 (26.24–28.97)	0.500
Putamen^§^	30.82 (2.03)	30.73 (2.70)	0.918
Caudate	31.84 (2.84)	31.78 (3.15)	0.948
Thalamus^*^	27.87 (26.65–29.18)	28.33 (27.32–29.12)	0.376

Note: ^*^Mann-Whitney U-test, numbers represent median. numbers in parentheses denote interquartile range; ^†^Younger (*n* = 10), older (*n* = 23); ^‡^Younger (*n* = 11), older (*n* = 24); ^§^ Younger (*n* = 13), older (*n* = 25); *P* < 0.05 are displayed in **bold. **$\:{T}_{\:\:2,f}^{ex}$ apparent relaxation time of relatively free water proton; $\:{T}_{\:\:2,r}^{ex}$ apparent relaxation time of relatively restricted water proton.

### CEST metrics

**Fig. 6:**
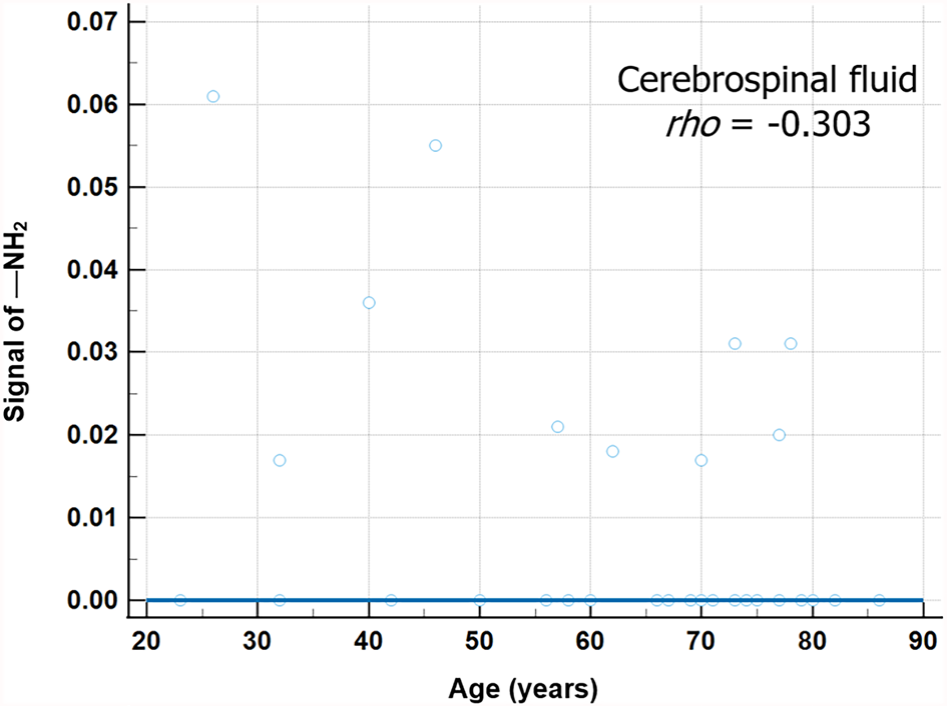
Correlation of —NH_2_ proton signal with age in CSF. Each blue circle represents the age of each participant (x-axis) and the mean signal of —NH_2_ proton (y-axis) within the CSF. The blue linear line shows the relation between the corresponding signals and ages. *rho* Spearman’s rank correlation coefficient.

The hydroxyl (—OH) proton peak was detected in most brain structures: 95.3% of (41/43) of cortical GM, 93.0% (40/43) of WM, 100% (43/43) of CSF, 86.0% (37/43) of hippocampus, 69.7% (23/33) of SN, 42.9% (15/35) of GP, 52.6% (20/38) of putamen, 48.9% (21/43) of caudate, and 81.4% (35/43) of thalamus. The amine (—NH_2_) proton peak was identified in 18.6% (8/43) of cortical GM, 23.3% (10/43) of WM, 23.3% (10/43) of CSF, 62.8% (27/43) of hippocampus, 42.4% (14/33) of SN, 17.1% (6/35) of GP, 10.5% (4/38) of putamen, 11.6% (5/43) of caudate, and 44.2% (19/43) of thalamus. The amide (—NH) peak was shown only in two older participants: in both the WM and GM of 77-year-old male, and in the WM of 78-year-old male. Among all CEST metrics, only the amine proton signal in CSF correlated with age (*rho =* −0.303; 95% CI, −0.553 to −0.003; *P* = 0.049) (Fig. 6) and differed between younger and older adults (median, 0 vs. 0; interquartile range, 0–0.025 vs. 0–0; *P* = 0.016, respectively). Detailed metrics for the comparison of two groups in all brain structures are provided in Table 4, and data comparison graphs of hydroxyl-related signal are presented in Supplementary Fig. 1.

**Table 4:** Comparison of median (interquartile range) of —OH and —NH_2_ between younger (< 65 years, *n* = 13) and older (≥ 65 years, *n* = 30) adult groups in multiple regions of interest.

Brain structure	Younger	Older	*P* value
—OH			
Cortical GM^*^	0.065 (0.043)	0.069 (0.034)	0.748
WM^*^	0.043 (0.021)	0.056 (0.029)	0.164
CSF	0.167 (0.109–0.197)	0.158 (0.115–0.176)	0.791
Hippocampus	0.062 (0.049–0.131)	0.082 (0.029–0.125)	1.000
Substantia nigra^†^	0.034 (0–0.065)	0.072 (0.008–0.081)	0.095
Globus pallidus^‡^	0.026 (0–0.033)	0 (0–0.050)	0.875
Putamen^§^	0.054 (0–0.060)	0 (0–0.037)	0.115
Caudate	0 (0–0.039)	0.009 (0–0.032)	0.977
Thalamus	0.059 (0.030–0.084)	0.067 (0.055–0.083)	0.499
—NH_2_			
Cortical GM	0 (0–0.005)	0 (0–0)	0.483
WM	0 (0–0.020)	0 (0–0)	0.486
CSF	0 (0–0.025)	0 (0–0)	**0.016**
Hippocampus	0.039 (0.028–0.070)	0.033 (0–0.079)	0.497
Substantia nigra^†^	0 (0–0.026)	0 (0–0.057)	0.617
Globus pallidus^‡^	0 (0–0))	0 (0–0)	0.914
Putamen^§^	0 (0–0)	0 (0–0)	0.488
Caudate	0 (0–0)	0 (0–0)	0.553
Thalamus	0 (0–0.006)	0 (0–0))	0.141

Note: ^*^Independent sample t test, numbers represent mean values. numbers in parentheses denote standard deviation; ^†^Younger (*n* = 10), older (*n* = 23); ^‡^Younger (*n* = 11), older (*n* = 24); ^§^ Younger (*n* = 13), older (*n* = 25); *P* < 0.05 are displayed in **bold.**

## Discussion

To the best of our knowledge, this study is the first to apply the CEST/ZAP technique in human brain to investigate whether multiparametric CEST/ZAP can capture region-specific aging changes in the human brain and can serve as potential biomarkers for aging-related brain alterations. We observed age-related changes in multiple CEST/ZAP metrics. WM showed significant increases in both F_f_ and $\:{T}_{\:\:2,f}^{ex}$ *(rho =* 0.482, 0.359, respectively; all *P* ≤ 0.018), whereas cortical GM exhibited significant positive correlations with age for $\:{T}_{\:\:2,f}^{ex}$ (*rho* = 0.432, *P* = 0.004). Deep GM structures demonstrated region-specific changes: the hippocampus, SN, GP, and putamen were associated with an age-related increase in F_f_ *(rho =* 0.347, 0.472, 0.538, and 0.371, respectively; all *P* ≤ 0.022), while the thalamus showed a significant increase in $\:{T}_{\:\:2,f}^{ex}$ (*rho =* 0.421; *P* = 0.005). In the CSF, age was associated with an increase in F_f_ *(rho =* 0.313, *P* = 0.041) in the ZAP metrics, accompanied by a decrease in amine-related signal (*rho =* −0.303; *P* = 0.049) in the CEST metrics. In the comparison between the two groups divided by 65 years of age, ZAP-derived F_f_ exhibited significant group differences in all brain subregions (all *P* ≤ 0.025). In addition, cortical GM, WM, CSF and thalamus showed significant inter-group differences in $\:{T}_{\:\:2,f}^{ex}$ (all *P* ≤ 0.008). For the CEST metrics, amine signal in CSF was significant lower in the older adult group (*P* = 0.016). Detectable amide signals were observed only in two older participants, either in WM alone or in both WM and GM, although no statistically significant group differences were found.

The increase in F_f_ with aging implies decreased macromolecular binding and increased water mobility, and it can reflect an increased amount of free water protons that are relatively unrestricted by their local microenvironment. Aging is associated with impaired water homeostasis, which may partly reflect dysfunction of the glymphatic system. These changes may involve increased interstitial fluid volume and altered expression or function of aquaporin channels, which may further influence water mobility and relaxation behavior^18^. The age-related increase in $\:{T}_{\:\:2,f}^{ex}$ may reflect an expansion of extracellular space due to microstructural changes, such as breakdown of structural barriers and decreased cell density, and increased interstitial or extracellular water, which permits greater mobility of free water molecules and prolonged transverse relaxation. In contrast, restricted water protons are more tightly associated with macromolecular structures like myelin, cellular membranes, and intracellular components^19,20^,^21^. An increase in $\:{T}_{\:\:2,r}^{ex}$ can be related to subtler changes in intracellular environments, such reduced macromolecular content, alterations in the microstructures of axons, or increased water mobility within restricted compartments^19,20,21^. In summary, these age-related changes in ZAP parameters may reflect decreased tissue integrity and changes in microenvironmental composition.

Degeneration and decreased myelination are key features of brain aging. Particularly, the WM deterioration is one of the most prominent alterations in the aging brain. A study reported a 15% loss of WM volume and a 27% reduction in the total length of myelinated nerve fibers^22^. As brain aging, oligodendrocytes (myelin-producing cells) and oligodendrocyte progenitor cells showed degenerations^23^, and it leads increased myelin breakdown, decreased remyelination, and fluctuations in the constituents of myelin and finally disruptions in WM integrity^24^. WM glial cells, such as astrocytes and microglia, play a critical role for preserving integrity and normal function of the WM. However, they can increase inflammation reaction with production of free radicals^25^, and decrease in the ability to remove toxic molecules from the extracellular medium in aging brain^26^. These cellular and microstructural change affects local microenvironment, leading to increase in F_f_ and $\:{T}_{\:\:2,f}^{ex}$. Regarding diffusion MRI-derived free water, increase of free water is observed in the WM during normal brain aging^27^. Also, several studies found that T2 relaxation times in the WM increase with age in regular proton MRI or non-exchange protons^19,28^. Their increase in non-exchange proton T2 relaxation times in WM may not directly be related to our increase in exchange free proton fraction; however, the exchangeability of exchange protons may be lost to non-exchange protons in altered environments, such as deformation of macromolecules, conformational change in macromolecules, and loss of boundary functions between exchange and non-exchange protons.

On the other hand, cortical GM and subregions of each deep GM showed a little different result from that of WM regarding ZAP metrics. There was a significant age-related increase in F_f_ in hippocampus, SN, GP, and putamen, and positive correlation between age and $\:{\mathrm{T}}_{\:\:2,\mathrm{f}}^{\mathrm{e}\mathrm{x}}$ in cortical GM and thalamus. Although most of myelinated fibers are present in the WM, GM contains significant amount of them^29,30^. In a longitudinal study using fast macromolecular proton fraction (MPF) mapping, age-related decrease in MPF were shown in GM including basal ganglia, hippocampus, as well as WM 31. We observe similar decreases of F_r_ with increasing ages, as F_f_ increases (F_f_ and F_r_ =1[16]) with ages in hippocampus, SN, GP, and putamen. Age-related demyelination or degradation of intracortical myelin may alter the local magnetic environment. However, such age-related increases of F_f_ were not shown in cortical GM, caudate and thalamus. These results may reflect mechanism beyond demyelination, potentially arising from difference in the tissue composition, microstructural organization, or intracellular environments between GM and WM.

Cortical thinning is a well-known aging process in the GM. Cortical thinning is related to a decrease in the complexity of dendrite arborization and regression the dendritic spines of pyramidal neurons^32,33^. Increase in the number and size of astrocytes and microglia^34^ was also associated with cortical thinning in aging brain: failure or dysregulation of upstream microglia and astrocyte cellular processes could lead to dendritic and synaptic changes and subsequent loss of cortical thickness^35^. These different cellular and microstructural changes between WM and GM lead to different results. Moreover, previous research demonstrated age-related iron deposition in almost all GM structures, although the results were different in subregional analyses^36–38^. Iron can have complex effects on water compartments and ZAP metrics. Increased iron content causes local magnetic field inhomogeneities that markedly shorten the apparent T2 relaxation time of both free- and restricted water protons^39,40^. At the same time, neurodegenerative and vascular processes linked to iron accumulation enlarge the extracellular space^40^, thereby increasing the fraction of free water protons. Although free water normally exhibits long T2, the local susceptibility effects in iron-rich regions partially counteract this, resulting in a net signal that reflects both elevated free water fraction and T2 shortening. This dual mechanism can explain the reason that GM shows different age-related change in ZAP metrics.

CSF showed an age-related increase in F_f_ in ZAP, along a reduction in signal from amine proton in CEST. According to aging and neurodegeneration, excessive efflux metabolites such as beta amyloid from interstitial fluid to ventricles can increase toxicity to CSF^41^, and choroidal plexus tissue suffers from oxidative injury and disrupted metabolism^42^. These pathological disturbances can enhance choroidal plexus permeability, leading to macromolecule accumulation. Finally, increase the osmotic increasing pressure of CSF could draw water into the ventricles^42^. Also, CSF space expands due to parenchyma atrophy in aging brain. These aging processes can affect ZAP results in current study. The major constituent of CSF is water (approximately 99%) with the remaining fraction composed of protein, glucose, ions, vitamins, and neurotransmitters^43^. Although age-related change in the composition of CSF have not been fully investigated, the production of some proteins decreases with age^44,45^, as well as age CSF to plasma ratios of multiple proteins are altered^46^. This biochemical alteration may contribute to the observed age-related deduction of amine signal in the CSF.

Amide proton signals were detected in only two middle-old participants (≥ 75 years). In a previous APT-weighted MRI study using a rat model of traumatic brain injury, increased APT-weighted signal was observed in perilesional edema, which was attributed to enhanced glial responses. Conversely, reductions in APT-weighted signal after neuroprotective treatment were associated with decreased microglial and astrocyte activation^47^. Impaired regulation of microglia and astrocyte can promote neuroinflammation, leading to mild acidosis and protein degradation in affected regions, both of which can influence amide signal^14^. Accordingly, the detection of amide proton signals in our older participants may reflect neuroinflammation processes associated with age-related neurodegenerative changes.

This study should be interpreted considering several limitations. First, although the age range of participants was broad (23–86 years), our study included relatively fewer individuals younger than 65 years old (*n* = 13) compared with those same or older than 65 years (*n* = 30). As a result, our findings may not fully capture the trajectory of alterations that occur in young adults. Second, not all CEST/ZAP metrics for the SN, GP, and putamen could be obtained for every participant due to incomplete anatomical coverage or difficulties in delineating reliable regions of interest (ROIs). Furthermore, these regions are particularly susceptible to iron accumulation with aging, which may complicate the results of ZAP metrics. Future research incorporating quantitative susceptibility mapping acquired concurrently may facilitate a more comprehensive evaluation of iron-related influences on ZAP metrics. Third, this study was also limited by the restricted slice coverage of the CEST/ZAP acquisition, which did not allow whole-brain analysis. Future studies with full-brain CEST/ZAP coverage may facilitate the use of comprehensive automated segmentation approaches and systematic region-wise assessment. Fourth, this study employed a single center, cross-sectional design with healthy volunteers, which limits the ability to infer causal relationships or characterize temporal trajectories of the observed changes. For instance, although amide proton signals were observed only in two older participants in our cohort, studies with larger sample sizes and inclusion of individuals with neurological disease may provide more robust and generalizable insights. Future longitudinal and multi-center studies, ideally incorporating clinical and cognitive assessments, will be required to establish the reliability and translational relevance of CEST/ZAP metrics in aging and neurodegeneration. Last, it should be noted that the ZAP framework provides a phenomenological representation of the Z-spectrum and does not explicitly model exchange kinetics as in quantitative magnetization transfer (qMT) approaches on Bloch–McConnell equations. Consequently, ZAP-derived parameters should be interpreted as effective descriptors of proton environments rather than direct measurements of specific exchange rates.

In conclusion, we demonstrated that multiparametrics derived from the combined CEST and ZAP techniques can identify microstructural and biochemical changes associated with aging across cortical and deep GM, WM, and CSF. These findings indicate that CEST/ZAP-derived metrics hold promise as noninvasive biomarkers for brain aging. Further research is warranted to validate the clinical utility of combined CEST and ZAP imaging in neurodegenerative diseases such as Alzheimer’ dementia and Parkinson’s disease and establish their role in earlier detection and longitudinal monitoring of neurodegenerative processes.

## Methods

This human subject study was approved by the Institutional Review Board of the University of California, San Diego (IRB No. 200335; approved on June 3, 2024). All procedures performed in this study involving human participants were compliant with the regulations of the Health Insurance Portability and Accountability Act (HIPAA). Written informed consent was obtained from all the study participants.

### Participants

A total of 43 consecutive healthy volunteers (24 females and 19 males; mean age 65.6 ± 16.0 years; age range 23–86 years) underwent brain MR imaging including combined CEST and ZAP protocol between June 2024 and June 2025. All participants were self-reported to be healthy with no history of neurological disease, and no significant structural abnormalities were observed on the whole-brain MRI using MPRAGE images. Thirty participants were same or older than 65 years and others (*n* = 13) were younger than 65 years.

### MRI Data Acquisition

All imaging studies were performed using a clinical 3 T scanner (Vantage Galan 3 T, Canon Medical Systems Corp, Japan), and 32 channel head coils (SPEEDER, Canon Medical Systems Corp, Japan).

Imaging sequence was multi-slice 2D single-shot fast-spin-echo (SSFSE) using the following parameters: repetition time (TR)/effective echo time (TE_eff_) = 1500/60 ms, flip/refocusing flip angles = 90°/160°, number of excitations = 1, matrix size = 256 × 256, field of view = 250 × 250 mm^2^, and three 5-mm thick axial slices with a 1-mm gap to cover the thalamus region. An MT preparation module consisted of 20 narrowband 40-ms sinc pulses with B_1_^rms^ = 2.0 𝜇T covering − 800 ppm to + 800 ppm range of offset frequencies with 56 points (Table 5). The offset frequency sampling followed a centric pattern, initiated at the water resonance (0 Hz) and symmetrically extended toward both positive and negative frequency offsets. This acquisition scheme enabled the use of a shorter repetition time (TR = 1500 ms) compared with TR = 10,000 ms required for full magnetization recovery in the original implementation^16^ with sequential offset frequencies sampling pattern, thereby reducing the total acquisition time for 56 offsets from over 9 min to approximately 84 s per slice. The total acquisition time for the three-slice scan is 4 min and 20 s, which may be acceptable for incorporation into clinical protocols. These three slices can cover most of the clinically relevant deep GM regions, including the hippocampus, thalamus, and basal ganglia. While CEST imaging covers range of offset frequencies from ± 8 ppm, the range of the ZAP frequencies is expanded to over ± 800 ppm. Denser sampling was implemented for the range of ± 1.3 kHz (− 10 to 10 ppm, comprising CEST range). This approach allowed to capture both the broader context and the finer details of chemical exchange processes.

**Table 5:** Frequency offset steps (Hz) and number of points at each step.

Offset step (Hz)	No. of points in “+” direction	No. of points in “-” direction
0	1	
50	11	11
300	1	1
3000	13	13
30,000	2	3
Total points	56	

Note: No. number.

To draw the ROI more accurately, T2-weighted image (T2WI) was acquired as a reference image, using the same slice levels as the CEST/ZAP. The Imaging sequence was fast advanced spin echo using following protocol: TR/TE = 3500/60 ms, number of excitations = 1, matrix size = 256 × 256, field of view = 250 × 250 mm^2^, three 5-mm thick axial slices with a 1-mm gap. The T1-weighted images (T1WI) were acquired to cover the same anatomical region and corresponding slice locations as the CEST/ZAP acquisition, although with a different spatial resolution and imaging matrix. This acquisition was not intended for full-brain coverage, but for tissue segmentation and subsequent registration to the CEST/ZAP data. Also, the T1WI were visually inspected for any structural abnormalities in the brain. We acquired sagittal 3D whole-brain MPRAGE with 1-mm isotropic resolution using following protocol: TR/TE/inversion time (TI) = 7.5/2.3/900 ms, flip angle = 9°, number of excitations = 1, matrix size = 288 × 288, field of view = 252 × 252 mm^2^, 1-mm thick sagittal slices with no gap, reformatted to axial and coronal views as well.

### Data analysis

Prior to the analysis, B_0_ correction was performed for each of ROI^48^. A two-Lorentzian compartment model was used for a voxel-by-voxel analysis. A dedicated neuroradiologist with 18 years of experience drew multiple ROIs in the hippocampus, SN, GP, putamen, caudate, and thalamus on T2WI using Horos (Horos Project, horosproject.org). It was not possible to draw ROIs in all anatomical structures depending on slice level, and such regions were skipped (10 SN, 8 GP, and 5 putamen). Following the previous work^15^, all data processing was implemented and conducted using in-house software in MATLAB (R2024a; MathWorks, Natick, MA, USA). The WM, cortical GM and CSF was automatically segmented using previously proposed method^49^. To apply the tissue segmentation masks to the CEST/ZAP data, a multi-step image alignment procedure was performed. Initial spatial correspondence between the T1WI and CEST/ZAP data was established using DICOM header information, including image position, orientation, voxel size, and direction cosine matrices. An affine multimodal registration was then performed in MATLAB (imregtform) between the T1WI and the ZAP image acquired at the largest offset frequency, using a Mattes mutual-information similarity metric optimized with a regular-step gradient-descent algorithm in a multi-resolution framework. The resulting affine transformation was applied to the WM, GM, and CSF masks to map them into the CEST/ZAP image space.

### Statistical analysis

Spearman’s rank correlation coefficient was used to evaluate the associations between age and ZAP metrics, and between age and CEST metrics. We divided the participants into two groups based on age 65 years: younger- (< 65 years, *n* = 13, 44.9 ± 13.9 years) and older (≥ 65 years, *n* = 30, 74.6 ± 4.8 years) adult group, and also conducted independent sample t- or Mann-Whitney U-tests, depending on whether the assumption of normality was satisfied for each group. When there are ROIs bilaterally, we take their average value, and when there is an ROI unilaterally, we take that value as is. Statistical analysis was performed using MedCalc^®^ v23.1.7 (Mariakerke, Belgium). *P* values < 0.05 were considered statistically significant.

## Supplementary Information

Below is the link to the electronic supplementary material.

Supplementary Material 1

Supplementary Material 2

Supplementary Material 3

Supplementary Material 4

Supplementary Material 5

Supplementary Material 6

Supplementary Material 7

Supplementary Material 8

Supplementary Material 9

## Data Availability

Data used and generated during this study are private and not publicly available but could be made available upon reasonable request.
