# Supplementary figures and images for: Multiparametric CEST and Z-spectrum analysis proton (ZAP) as biomarkers of human brain aging
Source: Sci Rep. 2026 Apr 4;16:16215. doi: 10.1038/s41598-026-46623-6 (PMC13201529; doi:10.1038/s41598-026-46623-6)

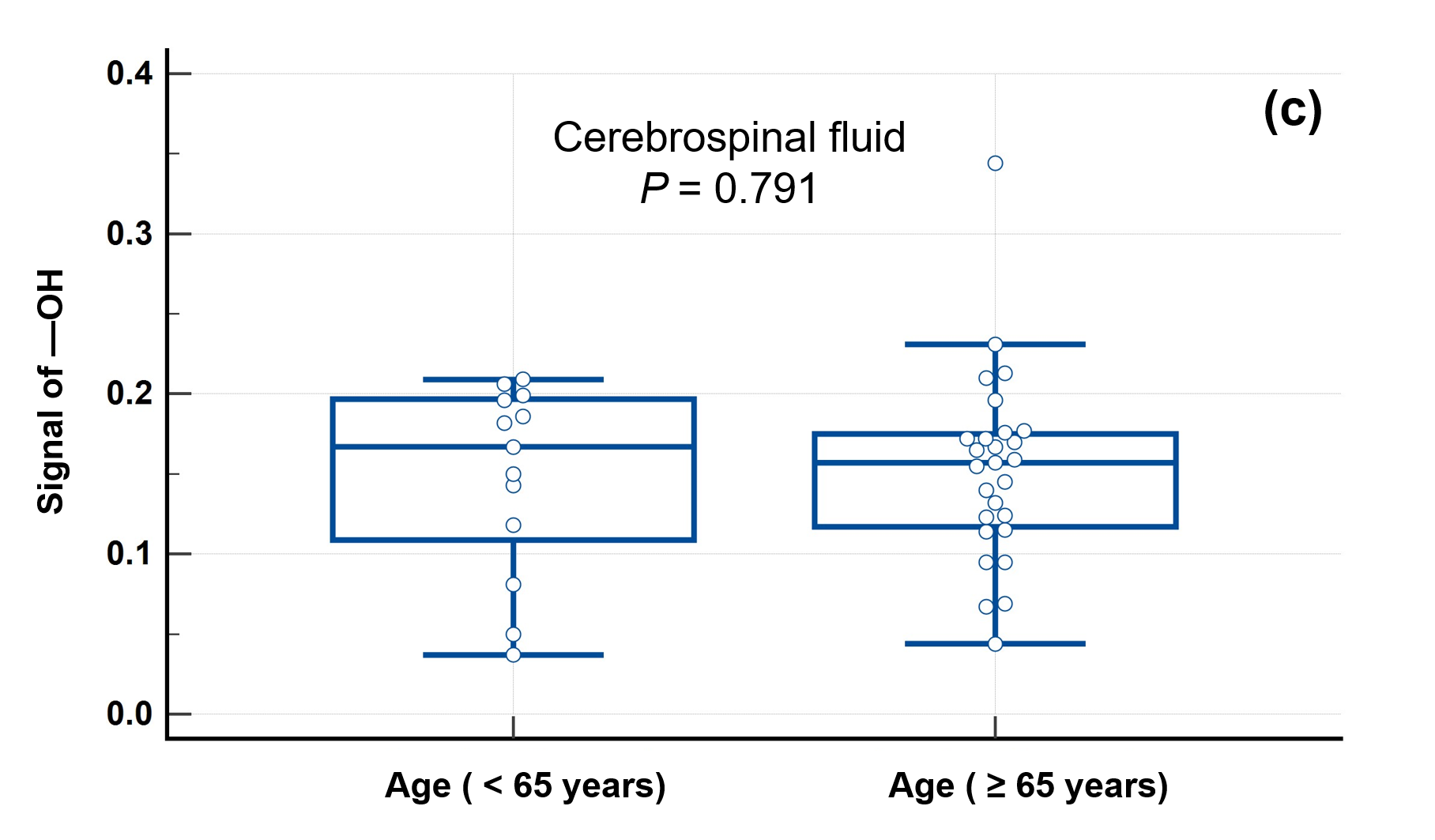

Supplement: Supplementary file 1 — Supplementary Material 1 [file 41598_2026_46623_MOESM1_ESM.tif]

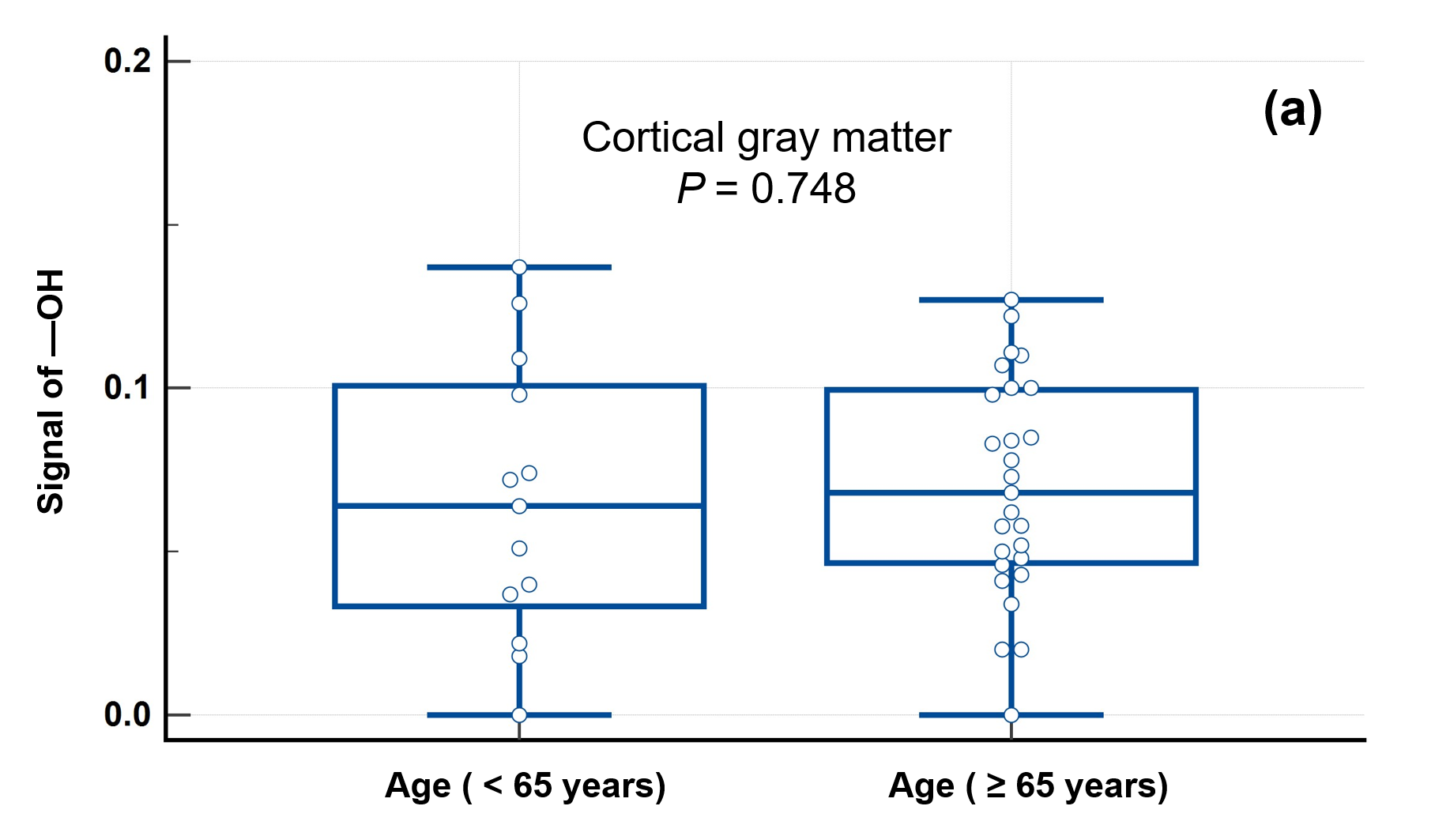

Supplement: Supplementary file 2 — Supplementary Material 2 [file 41598_2026_46623_MOESM2_ESM.tif]

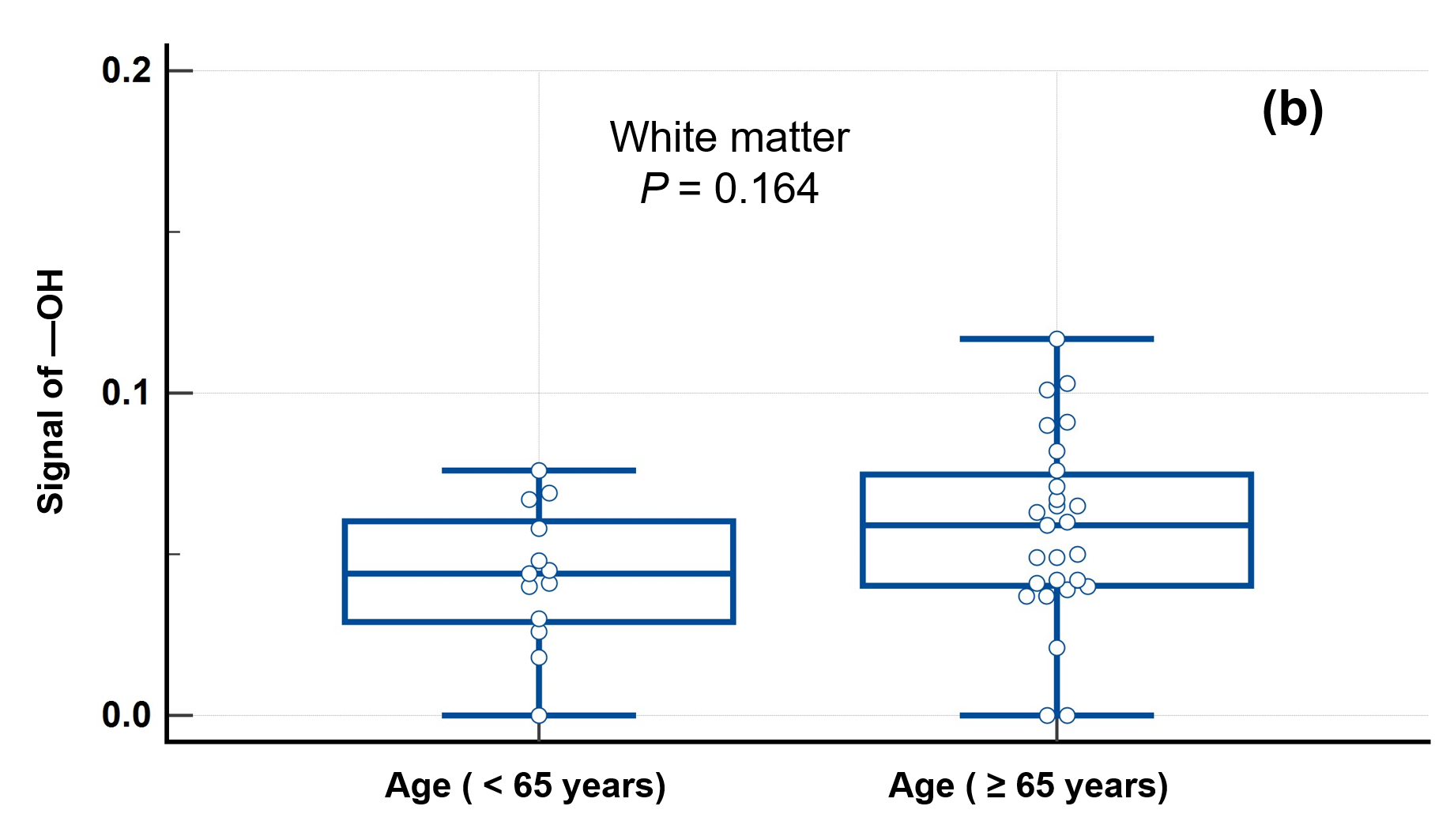

Supplement: Supplementary file 3 — Supplementary Material 3 [file 41598_2026_46623_MOESM3_ESM.tif]

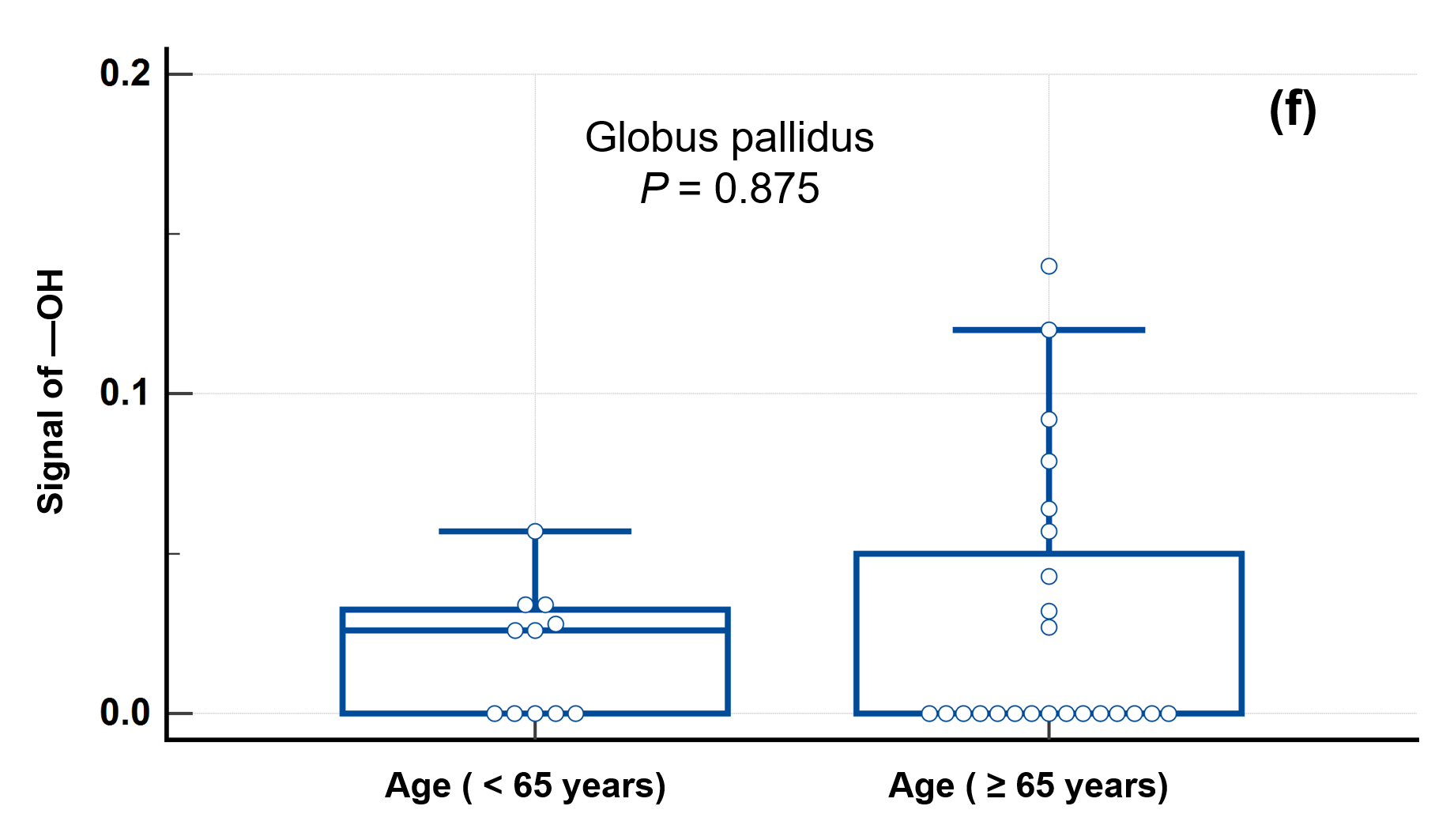

Supplement: Supplementary file 4 — Supplementary Material 4 [file 41598_2026_46623_MOESM4_ESM.tif]

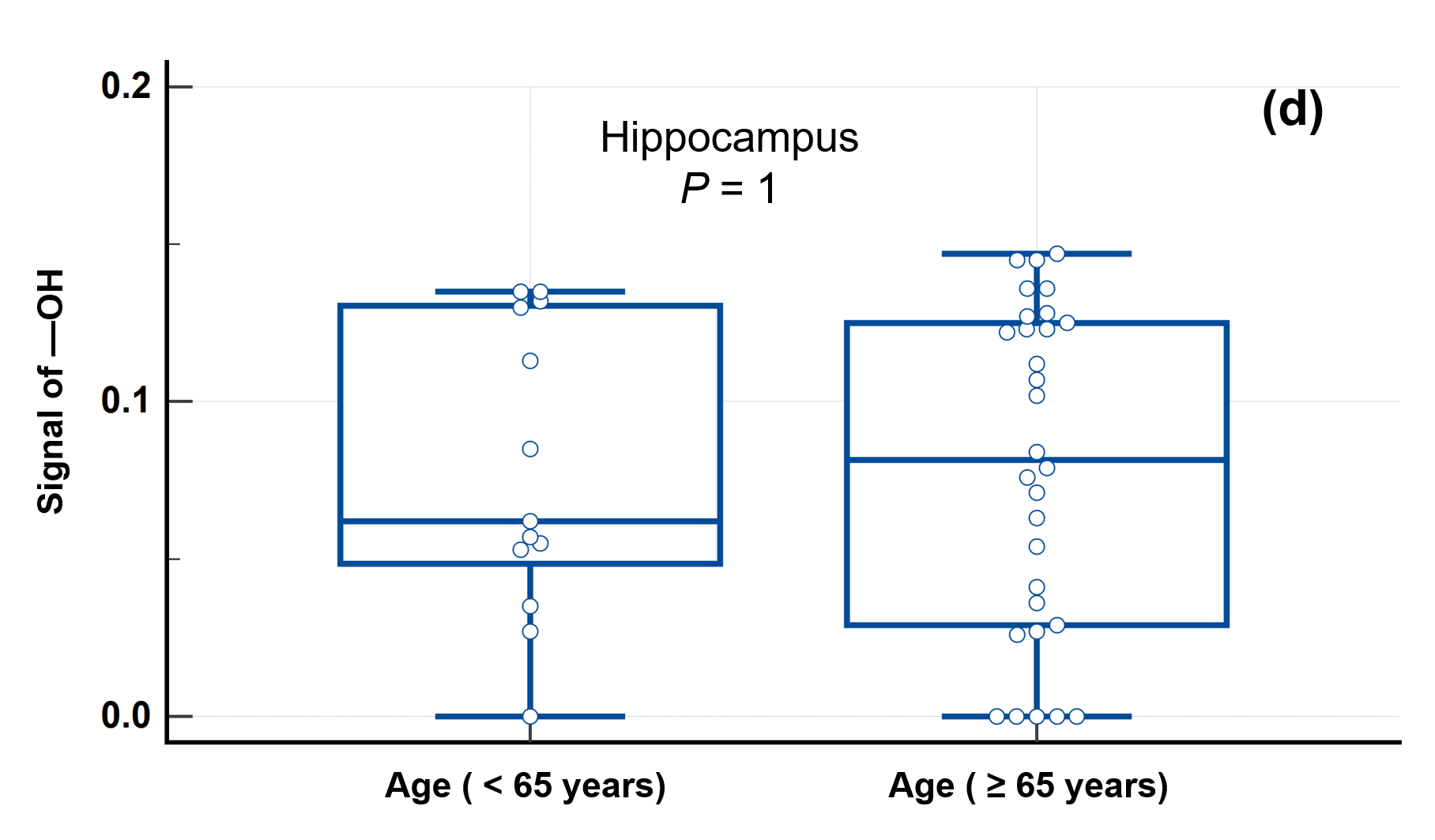

Supplement: Supplementary file 5 — Supplementary Material 5 [file 41598_2026_46623_MOESM5_ESM.tif]

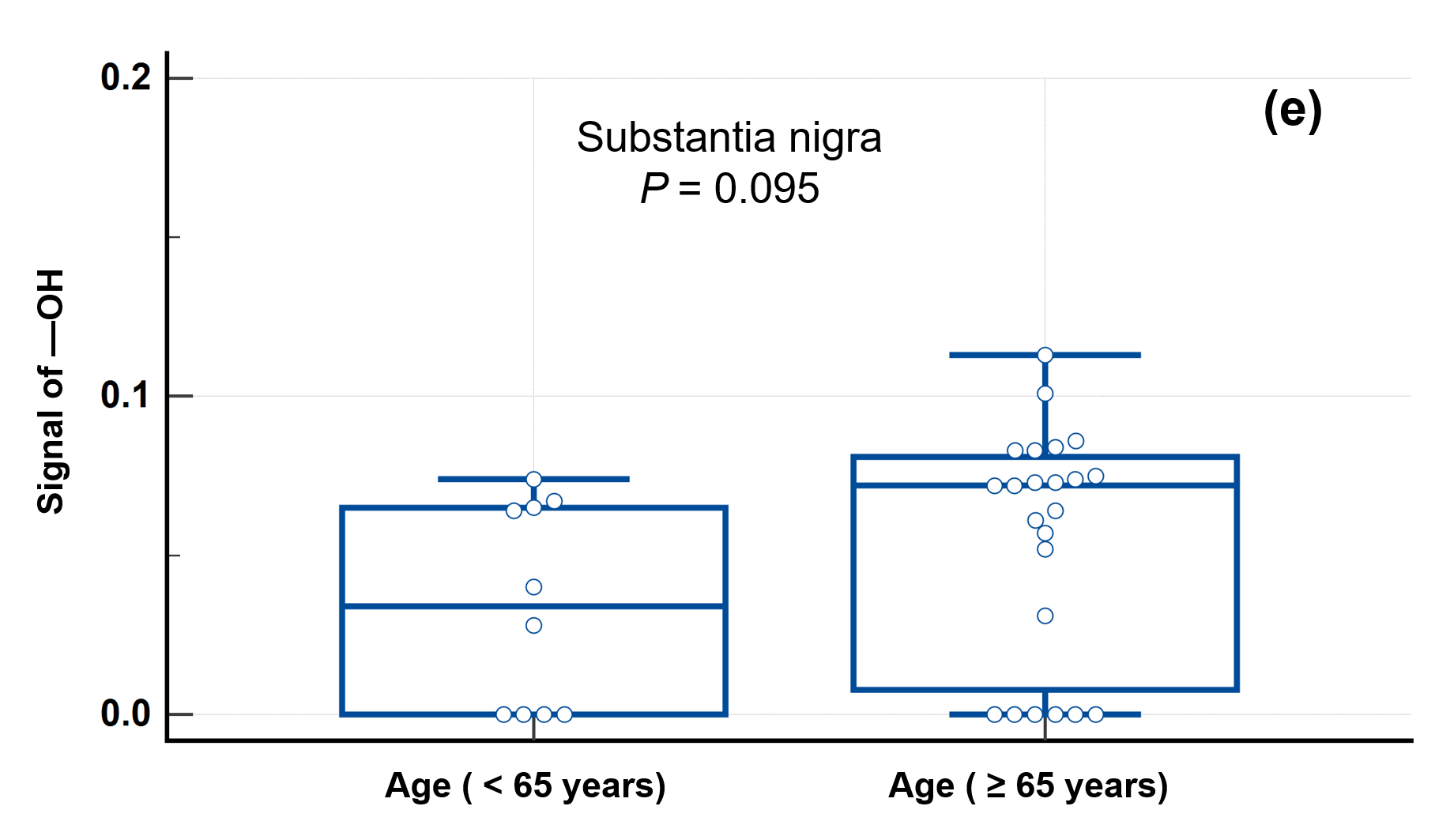

Supplement: Supplementary file 6 — Supplementary Material 6 [file 41598_2026_46623_MOESM6_ESM.tif]

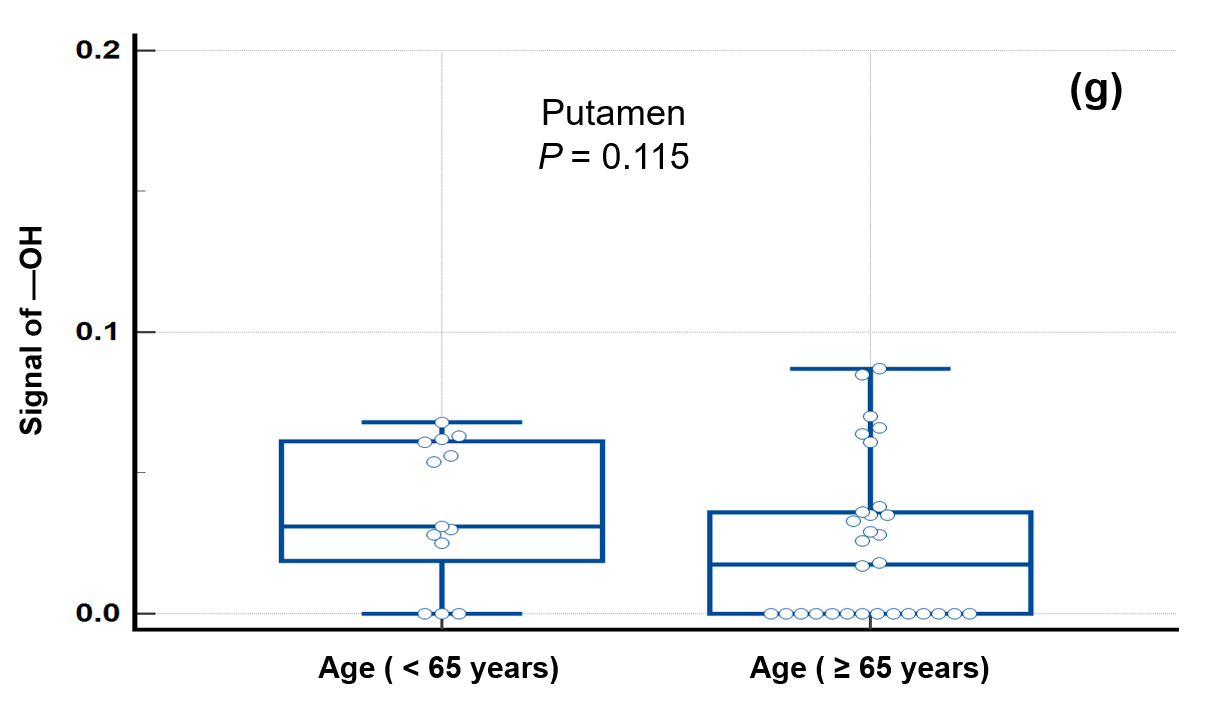

Supplement: Supplementary file 7 — Supplementary Material 7 [file 41598_2026_46623_MOESM7_ESM.tif]

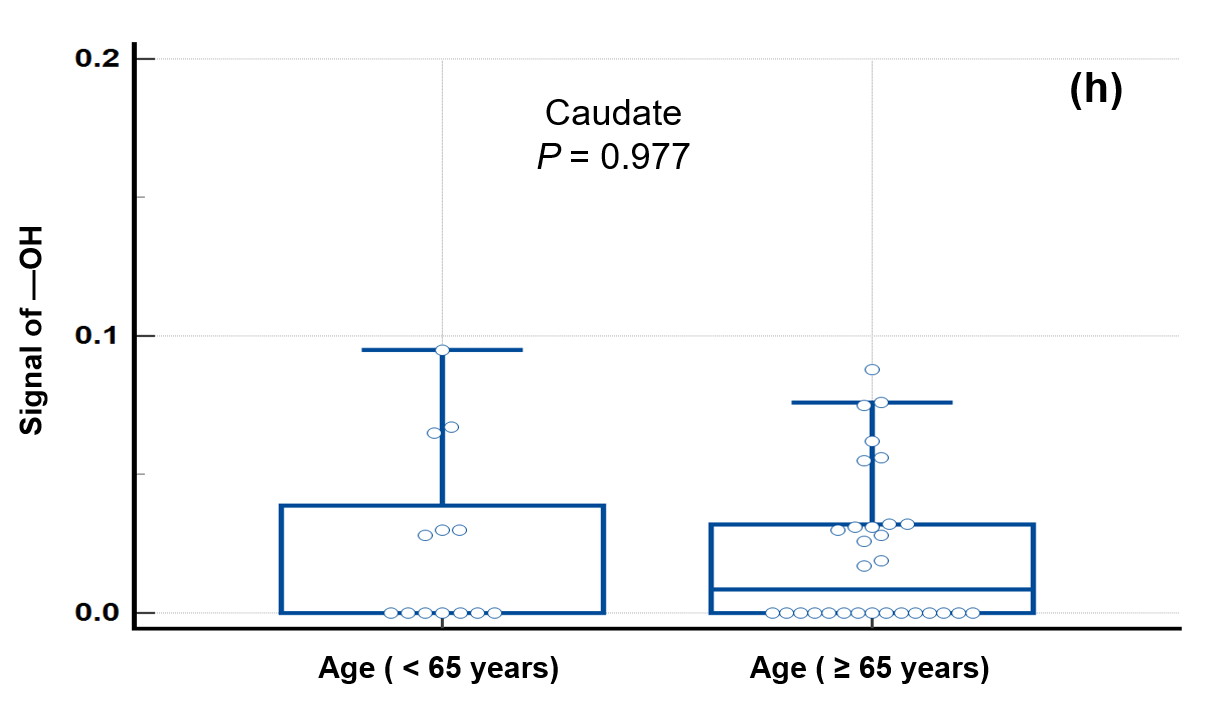

Supplement: Supplementary file 8 — Supplementary Material 8 [file 41598_2026_46623_MOESM8_ESM.tif]

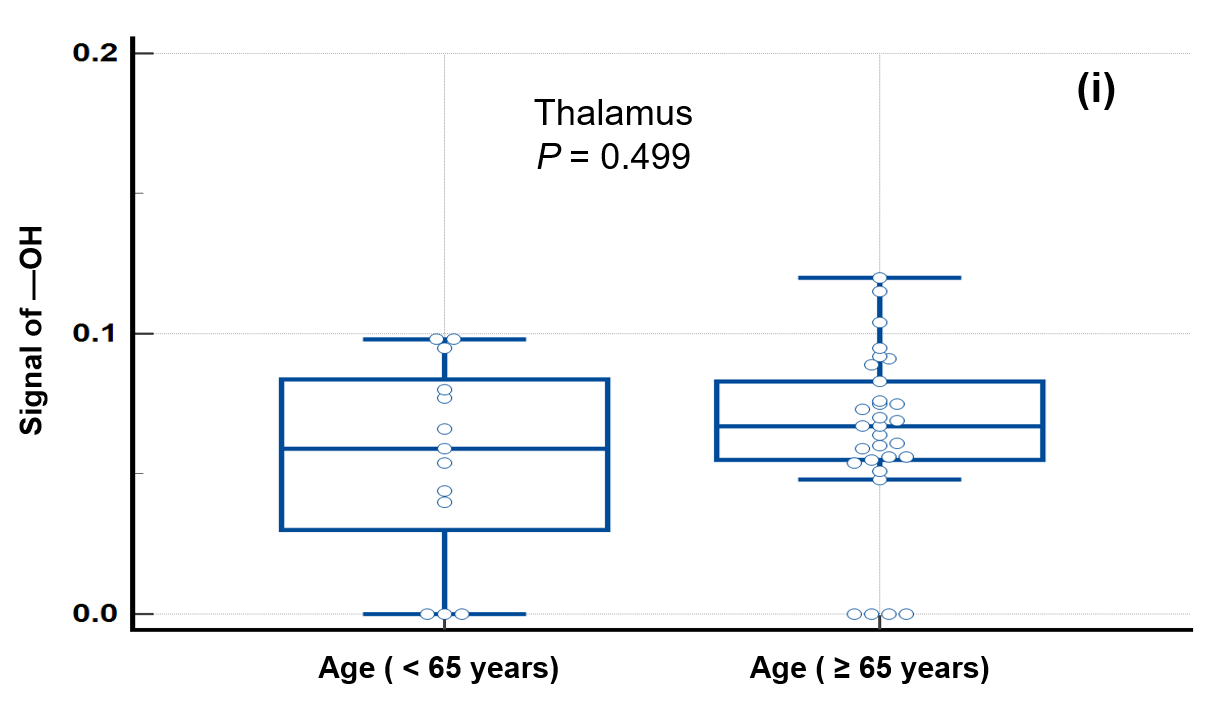

Supplement: Supplementary file 9 — Supplementary Material 9 [file 41598_2026_46623_MOESM9_ESM.tif]
